# Localized holes and delocalized electrons in photoexcited inorganic perovskites: Watching each atomic actor by picosecond X-ray absorption spectroscopy

**DOI:** 10.1063/1.4971999

**Published:** 2016-12-15

**Authors:** Fabio G. Santomauro, Jakob Grilj, Lars Mewes, Georgian Nedelcu, Sergii Yakunin, Thomas Rossi, Gloria Capano, André Al Haddad, James Budarz, Dominik Kinschel, Dario S. Ferreira, Giacomo Rossi, Mario Gutierrez Tovar, Daniel Grolimund, Valerie Samson, Maarten Nachtegaal, Grigory Smolentsev, Maksym V. Kovalenko, Majed Chergui

**Affiliations:** 1 Laboratoire de Spectroscopie Ultrarapide, ISIC-FSB and Lausanne Centre for Ultrafast Science (LACUS), Ecole Polytechnique Fédérale de Lausanne, CH-1015 Lausanne, Switzerland; 2 Department of Chemistry and Applied Biosciences, Institute of Inorganic Chemistry, ETH Zürich, CH-8093 Zürich, Switzerland; 3 Laboratory for Thin Films and Photovoltaics, Empa − Swiss Federal Laboratories for Materials Science and Technology, CH-8600 Dübendorf, Switzerland; 4 Paul Scherrer Institut, CH-5232 Villigen, Switzerland

## Abstract

We report on an element-selective study of the fate of charge carriers in photoexcited inorganic CsPbBr_3_ and CsPb(ClBr)_3_ perovskite nanocrystals in toluene solutions using time-resolved X-ray absorption spectroscopy with 80 ps time resolution. Probing the Br K-edge, the Pb L_3_-edge, and the Cs L_2_-edge, we find that holes in the valence band are localized at Br atoms, forming small polarons, while electrons appear as delocalized in the conduction band. No signature of either electronic or structural changes is observed at the Cs L_2_-edge. The results at the Br and Pb edges suggest the existence of a weakly localized exciton, while the absence of signatures at the Cs edge indicates that the Cs^+^ cation plays no role in the charge transport, at least beyond 80 ps. This first, time-resolved element-specific study of perovskites helps understand the rather modest charge carrier mobilities in these materials.

## I. INTRODUCTION

Perovskites are a class of crystalline materials with the formula ABX_3_, in which A and B are cations and X represents an anion. Lead-halide organic-inorganic perovskites (in which A is a monovalent organic cation) have recently emerged as highly promising optoelectronic materials for photovoltaics, photodetection, light-emitting diodes, and laser devices.^1–8^ Solar cells with lead-halide organic-inorganic perovskites (e.g., CH_3_NH_3_PbI_3_), prepared by low-temperature solution-based methods, have recently achieved outstanding performances with photo-conversion yields near 22%.^9,10^ This high conversion efficiency, together with the low processing temperature (150 °C), make them ideal candidates for the development of low-cost solar cells.

Despite the intense interest for organic-inorganic perovskites, a clear understanding of the fate of the photoinduced charge carriers remains limited. Lead-halide organic-inorganic perovskites exhibit a sharp absorption onset at the optical band edge, with a small extension of the Urbach tail (∼15 meV).^11,12^ The photoluminescence (PL) spectrum is near the gap (i.e., weakly Stokes-shifted) and is homogeneously broadened by interactions with phonons, leading to a considerable intensity beyond the band edge.^13^ Additionally, long carrier lifetimes and low non-radiative losses have been reported,^13,14^ pointing to a low density of traps (∼10^10^ cm^−3^ in single crystals)^15–17^ and leading to high PL quantum yields (>70%) for organic-inorganic perovskites.^14,18^ Nevertheless, the charge carrier mobilities in these perovskites are rather modest,^19^ and in the case of the electrons, at least 1 order of magnitude lower than in materials such as silicon or gallium arsenide.

In general, photovoltaic materials require efficient separation of photocarriers and the exciton binding energies (BE ≈ 10–70 meV), reported for organic-inorganic perovskites,^20–22^ are on the same order of magnitude as the room temperature (RT) thermal energy,^11,16,21^ raising the question whether the transport of energy occurs by free carriers or by a bound exciton that later dissociates into free carriers at heterojunctions.^23–25^

RT PL studies have been performed to address these questions, but both exciton recombination^26–29^ and free carrier recombination have been invoked.^20^ Recently, He *et al.* suggested that the RT PL is due to weakly localized excitons,^30^ which are due to band tail states, presumably arising from the disorder introduced by the organic cation. These excitons can be either partly localized (one charge carrier is localized with another carrier bound to it by Coulomb attraction) or fully localized (both charge carriers are localized).

Organic-inorganic perovskites have raised concerns about their long-term thermal stability under the working conditions of solar cells.^31–33^ Migration of the methyl ammonium cations (CH_3_NH_3_^+^) is a factor of degradation,^34^ while moisture can catalytically deprotonate them.^35^ These last two problems are minimized by replacing the organic cation by an inorganic one, such as caesium, as this leads to more stable materials under long term irradiation.^33,36–40^ Stoichiometrically, the inorganic CsPbBr_3_ material consists of one Cs^+^ and one Pb^2+^ cation for three halides (e.g., Cl^−^, Br^−^, or I^−^), as depicted in Figure S1 (supplementary material), forming a cubic crystal structure (Figures S2–S4, supplementary material). In the case of the mixed-halide CsPb(ClBr)_3_ material, the composition is of about 2 Br^−^ ions to one Cl^−^.

The physics of organic-inorganic perovskites and inorganic ones bear many analogies. Photoemission and inverse photoemission studies have shown that they have identical electronic structures.^41^ This is supported by calculations of the electronic band structure of CsPbBr_3_ and CsPbCl_3_,^36,41–43^ which show similar trends to organic ones.^8,44–48^ In these systems, the top of the valence band (VB) is dominated by the halide p-orbitals (Br 4p or Cl 3p), with a weak contribution of Pb 6s orbitals, while the bottom of the conduction band (CB) is dominated by the Pb 6p-orbitals, with a weak contribution of halogen p-orbitals.^36,43^ In both classes of perovskites, the orbitals of the cation (organic or Cs) are either far below the maximum of the VB or far above the minimum of the CB. Another common aspect to both types of perovskites is their high PL quantum yield, which reaches 90% in the inorganic case^36^ and 70% in the organic-inorganic case.^14,18^ Furthermore, from a dynamical point of view, transient absorption (TA) studies from the femtosecond to the nanosecond regime also point to a similar overall behavior of the charge carriers in organic-inorganic^49–51^ and inorganic ones.^52,53^ Finally, recent work has shown that solar cells made of mixed cation (Cs, formamidinium, and methylammonium) perovskites reach 20.8% conversion efficiency,^9^ while solar energy conversion has been demonstrated with purely inorganic Cs-based perovskites.^33,40^ Furthermore, the ease of tuning their band gap properties by controlling the halide stoichiometry^36,54,55^ holds great promise for successful use in tandem solar technologies.^33,37^ Therefore, due to their greater stability, their simpler structure, and ease of synthesis, inorganic perovskites represent the next generation of materials for solar energy conversion. In return, the study of the charge carriers in inorganic perovskites will shed light on their fate in organic-inorganic ones.

The description of charge carriers in inorganic or organic-inorganic perovskites requires tools that are site-sensitive and/or element-specific and can probe the time evolution of the system at RT. Electron paramagnetic resonance (EPR) has been used to determine charge localization in solar materials,^56^ but it requires low temperatures (LT). Shkrob and Marin.^57^ studied by EPR LT (<200 K) CH_3_NH_3_PbI_3_ and CH_3_NH_3_PbBr_3_ samples irradiated at 355 nm and reported localization of holes at the organic cations and of the electrons at Pb^2+^ centres, which formed clusters. This along with a change of color of the crystal surface during the measurements suggests that the samples were undergoing radiation damage. Besides, EPR lacks the time resolution that would help identify the formation and evolution of charge carriers (e.g., trapping at defects or via coupling to phonons).

In recent years, time-resolved (from 100 femtoseconds to 10s picoseconds resolution) X-ray absorption spectroscopy (TR-XAS) has emerged as an ideal tool for probing charge transfer processes in a wide variety of molecular, biological, and nano-systems.^58–60^ In particular, we recently implemented it to probe the electron trapping at defects in RT bare and dye-sensitized anatase TiO_2_ colloidal nanoparticles with picosecond^2^ and femtosecond^61^ time resolution. Photo induced Ti^3+^ traps were identified with those formed by electron delivery via inter-gap excitation distinguishable from those delivered by interfacial electron injection.

In this article, we investigate the fate of the charge carriers in photoexcited inorganic CsPbBr_3_ and CsPb(ClBr)_3_ perovskites by interrogating each atom of the material using time-resolved XAS with 80 ps resolution at the Br K-edge (near 13.474 keV), the Pb L_3_-edge (near 13.035 keV), and the Cs L_2_ edge (near 5.359 keV) perovskite nanocrystals (NCs).^36^ CsPb(ClBr)_3_ is a material with a band-gap energy and an exciton BE between those of the pure brominated and pure chlorinated Cs-based perovskites and is studied here in order to explore the role of halogen substitution on the fate of the charge carriers. Indeed, halogen substitution shifts in energy the VB more than the CB, as seen from calculations on organic-inorganic perovskites.^62^ Our results show that while for both systems, the hole is localized at Br atoms in the VB, electrons remain delocalized in the CB and Cs atoms exhibit no response to photoexcitation.

## II. EXPERIMENTAL PROCEDURES

The samples consisted of nanocrystals (NCs) in colloidal solution. The use of a free flowing liquid jet (200 *μ*m thickness) serves to minimize photo-damage by continuously refreshing the sample during the measurements. The time-resolved XAS experiments (more details in Sec. S2, supplementary material) were carried out using the high repetition rate scheme at the microXAS and SuperXAS beamlines of the Swiss Light Source (SLS).^63^ The pump laser ran at a repetition rate of 260 kHz delivering 10 ps-duration excitation pulses at 355 nm, which is above the band gap of the samples (Figure S5, supplementary material). The temporal width of the X-ray pulses is 80 ps at the SLS, which defines the time resolution of the experiment.

We recorded the UV-visible and XAS spectra before and after each measurement series (Figures S5 and S6, supplementary material), and they show no damage to the sample during the measurements (see Sec. S3, supplementary material). Finally, the pump fluence dependence of the transient Br K-edge signal (Figure S7, supplementary material) exhibits a linear dependence up to at least 15 mJ/cm^2^. Therefore, the latter fluence was used for the measurements.

The synthesis and characterization (transmission electron microscopy, X-ray powder diffraction, UV-visible spectroscopy, and XAS) of the perovskite NCs studied here are described in Sec. S1 (supplementary material) and in Ref. 36. The NCs involved in this work have an average size of ∼12 nm (Figure S2, supplementary material) and were suspended in toluene. Given that the exciton radius is between 5 to 7 nm,^36^ issues of quantum confinement are not expected.

## III. RESULTS

**FIG. 1.**
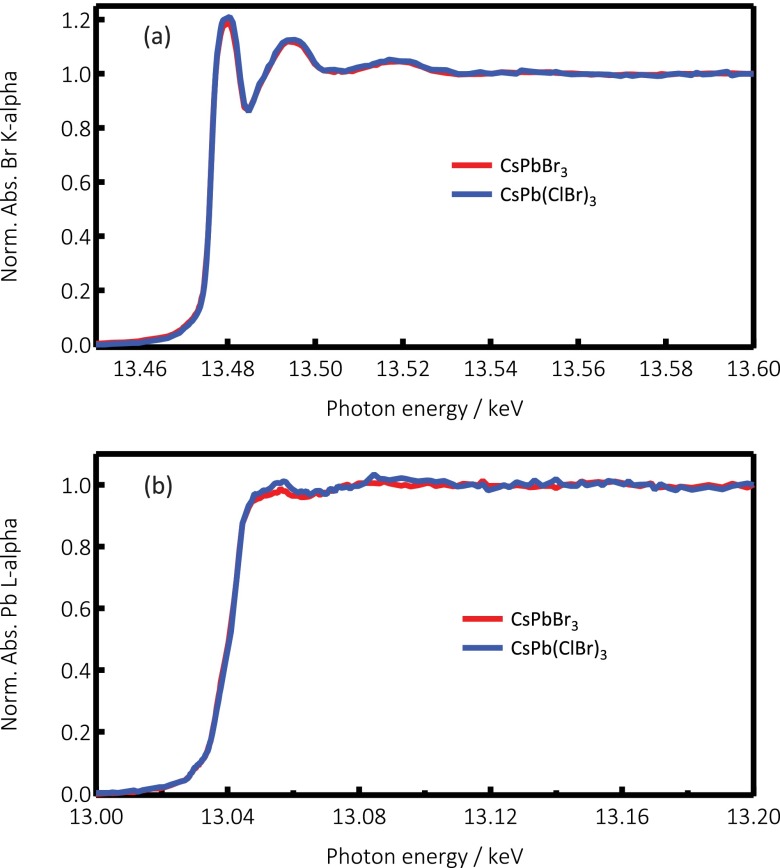
PFY X-ray absorption spectra near the edge region of CsPbBr_3_ (red trace) and CsPb(ClBr)_3_ (blue trace) NCs at the Br K-edge (a) and Pb L_3_-edge (b). The difference in intensity of the Pb L_3_-edges at about 13.05 keV between the two compounds is related to a higher density of unoccupied states due to the presence of Cl.

Figure 1 shows the steady state Br K-edge and Pb L_3_-edge absorption spectra of CsPbBr_3_ and CsPb(ClBr)_3_ NCs, detected in partial fluorescence yield (PFY) mode (see Sec. S.2, supplementary material). The Br K-edge spectra (Figure 1(a)) are somewhat different when recorded in total fluorescence yield (TFY) mode (see Sec. S.2), as can be seen in Figure S6 (supplementary material), but they resemble the spectrum reported for organic CH_3_NH_3_PbBr_3_ perovskites.^64^ Interestingly, the K-edge absorption spectrum of the Br^−^ ions in the present samples is quite similar to that of the aqueous Br^−^, also recorded in PFY mode.^65^ Since Br^−^ has a fully filled 4p^6^ valence orbital, the signal is entirely due to above-edge transitions into the ionization continuum with the X-ray absorption near-edge structure (XANES) modulations rapidly damping out.

**FIG. 2.**
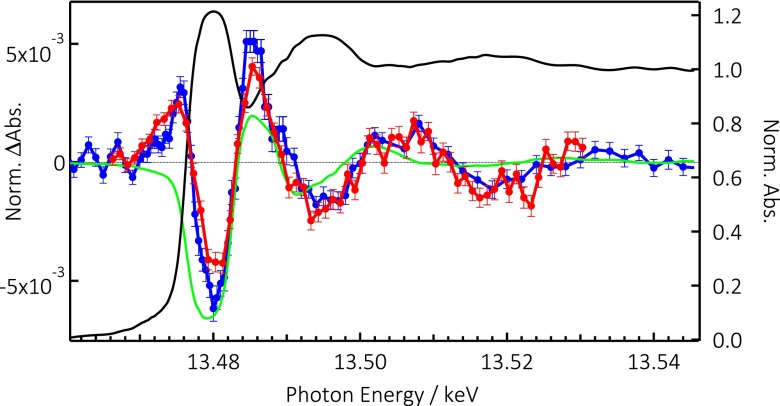
Partial Fluorescence Yield (PFY) X-ray absorption spectrum of CsPb(ClBr)_3_ at the Br K-edge (black trace) with transients at 100 ps of CsPb(ClBr)_3_ (blue trace) and CsPbBr_3_ (red trace) excited at 355 nm with 15 mJ/cm^2^. The transients were recorded in total fluorescence yield (TFY) detection mode. The green trace represents the difference of the steady state spectrum (black trace) shifted by +5 eV minus the unshifted steady-state spectrum.

Figure 2 also shows the transient Br K-edge spectrum (difference spectrum of the X-ray absorbance of the excited minus the unexcited sample) of CsPb(ClBr)_3_ and CsPbBr_3_, recorded 100 ps after laser excitation. The two transients exhibit the same profile, though with somewhat different amplitudes, showing an increased absorption below (around 13.475 keV) and above (around 13.485 keV) the edge and a decreased one at the edge (13.480 keV). At higher energies, clear modulations show up. As can be seen from Figure S8 (supplementary material), the energy transients are identical at 100 ps and 1 ns time delay, to within error bars.

**FIG. 3.**
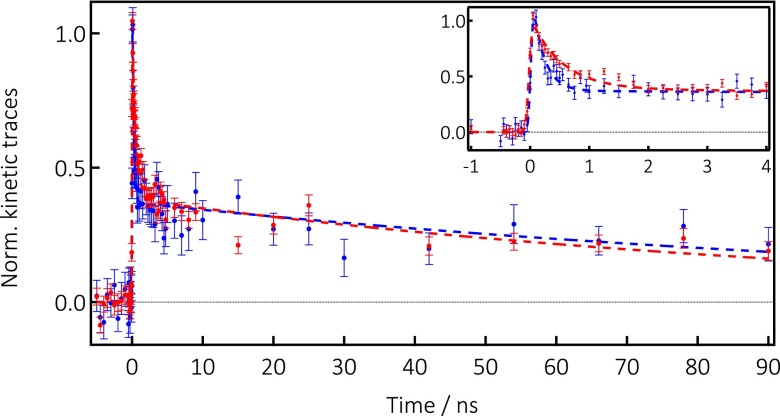
Kinetic traces of the X-ray signal at 13.048 keV upon photoexcitation at 355 nm of CsPb(ClBr)_3_ (blue dots) and CsPbBr_3_ (red dots) NCs using a fluence of 15 mJ/cm^2^. The dashed lines represent a bi-exponential fit (see Sec. S.10 (supplementary material)) yielding the fit parameters listed in Table I. The inset is a zoom of the first 4 ns after excitation.

The time evolution of the transient X-ray signal was recorded at 13.480 keV and is shown in Figure 3 for both samples. To within error bars, the time traces are quite similar, with an intensity drop within the first 3 ns, followed by a long decay component stretching over 100 ns. However, at sub-ns times, differences show up with a slower decay for the Cl-free sample (Figure 3). The similar kinetics of the signal (Figure S9, supplementary material) at the edge (13.480 keV) and above it (13.4863 keV) points to the spectral changes in Figure 2 belonging to the same transient species.

**FIG. 4.**
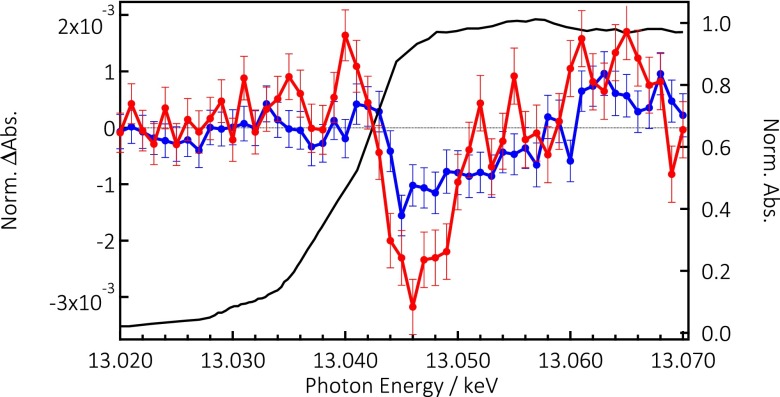
PFY Steady-state X-ray absorption spectrum of CsPb(ClBr)_3_ NCs at the Pb L_3_-edge (black trace) and transient spectra at 100 ps time delay after photoexcitation at 355 nm with 15 mJ/cm^2^ of CsPb(ClBr)_3_ (blue trace) and CsPbBr_3_ (red trace).

Figure 1(b) shows the steady state PFY Pb L_3_-edge spectra of both samples. They are identical, and are also quite similar to the Pb L_3_ spectrum reported^64^ for CH_3_NH_3_PbBr_3_ nanoparticles. They are overall rather featureless, which is typical of Pb L-edge spectra (Figure S10, supplementary material).^66,67^ Figure 4 shows the Pb L_3_-edge transients obtained for the two samples. The transients show some similar trends for both samples, bearing in mind the large error bars, due to higher noise: a decreased absorption right at the top of the edge (near 13.05 keV), while the signal appears positive above 13.065 keV. The signal also appears positive below the edge for the case of CsPbBr_3_. Due to the low signal, we could not record time traces.

**FIG. 5.**
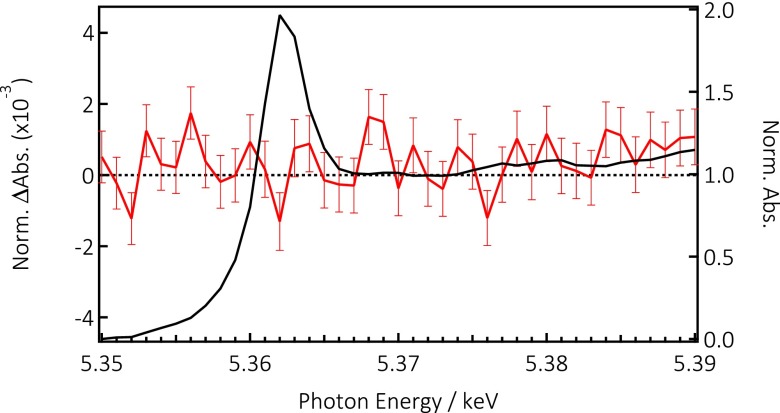
X-ray absorption spectrum of CsPbBr_3_ NCs at the Cs L_2_-edge (black trace) with the transient spectra at 100 ps (red trace) showing no signal within the S/N of the measurement.

Finally, Figure 5 shows the Cs L_2_-edge, which is characterized by a prominent “white line” at 5.363 keV, followed by a rather featureless above-edge region. Since Cs is in the +1 oxidation state, the white line edge is due to the 2p_1/2_ to 6s transition. Under 355 nm excitation, no photo induced changes are observed at the Cs L_2_-edge, contrary to the case of the Br and Pb edges. We therefore conclude that the Cs atoms are not affected by photoexcitation at times ≥80 ps and we discuss this observation below.

## IV. DISCUSSION

Generally speaking, in X-ray absorption spectra, oxidation state changes of an element show up as a shift of the edge position (i.e., the ionization potential for the atomic core orbital in question), which increases or decreases in energy depending, respectively, on whether the atom has been oxidized or reduced.^68^ However, the edge shift contains other causes, e.g., the type of ligand and the bond distance between the atom of interest and its neighbors.^69–71^ Photoinduced structural changes will therefore have a direct impact on the edge, even in the absence of an oxidation state change.^72,73^ Since the above-edge transitions probe the unoccupied density of states above the Fermi level, a reduction of intensity right above the edge reflects the filling of unoccupied states, similar to the band filling found in optical studies of semi-conductors.^74^

In the systems studied here, the VB is dominated by the halogen n*p* orbitals (in the present case, Br 4p or Br4p/Cl3p) and to a lesser extent, by the Pb 6s orbitals.^36,41,43,45,46^ The holes (electrons) created in the VB (CB) upon photoexcitation can either be delocalized or become localized at defects or a regular lattice site via a self-trapping process. In the case of delocalization, only a tiny fraction of the charge is “felt” by each atom and no edge shift can be detected. In the case of localization, the removal of a full or partial electron charge, e.g., the localization of a partial or full hole charge, turning the bromide to Br^0^(4p^5^) would lead to an edge shift to higher energies. In addition, this would open a channel for the 1s-4p transition that lies just below the edge. This behavior of the XAS transients has been reported after electron abstraction of photoexcited aqueous Br^−^ at the K-edge^65^ and aqueous I^−^ at the L_1_-edge.^75^ It is reproduced here with the resonance at 13.477 keV attributed to the 1s-4p transition, while the minimum at 13.48 keV and the maximum at 13.486 keV are typical of a first derivative-like shape due to a blue shift of the edge.

An estimate of the magnitude of the photo-induced blue shift of the Br K-edge (Figure 2) can be made by taking the difference of the blue-shifted ground state spectrum minus the unshifted spectrum. This approach works quite well, as demonstrated in the case of photo-ionized halides in aqueous solutions,^65,75^ and charge transfer in metal complexes.^58,76^ However, it neglects the other sources of edge shift mentioned above such as structural changes, nor does it account for the changes in the occupancy of orbitals below the edge, which affect the pre-edge (bound-bound) transitions. Bearing in mind these limitations, we calculated the difference spectra for shifts of +1 to +8 eV, which are compared to the experimental transient in Figure S11, supplementary material. In making these differences, we used the PFY spectrum (Figure 1(a)) because the wide energy range explored allows a fine pre- and post-edge normalization. However, since the transients were recorded in TFY mode (in Sec. S2, supplementary material), this may cause an additional source of deviation between the experimental transients and calculated difference spectra. Although in Figure S11 (supplementary material), none of the difference spectra satisfactorily matches the first derivative-like shape between 13.48 and 13.49 keV, the 5–6 eV shift is the best at capturing the 13.48 keV to 13.485 keV region (Figure 2). In the case of aqueous bromide turning to neutral bromine upon electron abstraction, Elles *et al.*^65^ reported an edge shift of ∼5 eV, close to the present value. We stress again that the deviations below 13.48 keV (appearance of the 1s-4p resonance) cannot be accounted for in the difference spectra. Also, the deviations above 13.485 keV are due to structural changes in the environment of Br^−^ ions after they have been oxidised. In particular, the Br K-edge has been reported to be very sensitive to and undergo significant edge shifts depending on its environment, even without a change of the oxidation state.^69–71^

The appearance of the 1s–4p resonance at 13.475 keV and the estimated oxidation shift of ∼5 eV both point to a full positive charge localizing at the Br centres.^65^ Charge localization may be caused by small hole polaron formation either at a defect or at a regular site of the lattice. In either case, the consequence of the charge localization is a bond elongation between the Br atoms, that have turned neutral, and their nearest neighbors. We suggest that formation of small polarons at regular sites is the main outcome of the photoexcitation, based on the following: (a) The transient exhibits rather clear modulations above the edge. Since defects exist in a distribution of geometries, charge trapping at these defects would lead to a washing out of the above-edge modulations, as was the case in our previous study on anatase TiO_2_.^2^ The fact that the transient shows a clear-cut modulation above the edge points to the excited charge carriers being localized at well-structured sites, which are most likely regular Br-sites of the lattice; (b) Furthermore, the high quantum yield of 90% for the band gap PL also points to the overwhelming majority of charge carriers being in the regular lattice, rather than at defects. The localization of a full positive charge at Br atoms of the regular lattice implies formation of a small hole polaron via coupling to phonons. Theoretical simulations are planned to confirm this hypothesis.

The kinetic traces in Figure 3 were phenomenologically fit using a bi-exponential function convoluted to the instrument response function of 80 ps, and the time constants and pre-exponential factors are given in Table I. While the long component is almost identical in both cases (100–130 ns), the short one increases by a factor of 2.5–2.7 from CsPb(ClBr)_3_ to CsPbBr_3_. Our purpose here is not to interpret these timescales, which require further studies but we stress the analogy with the RT PL decay kinetics of organic-inorganic perovskites. Indeed, a biexponential decay has been reported for the latter,^9,14,28,30,77,78^ with the short component usually on the order of a few nanoseconds, and the long one spanning from tens to hundreds of ns. The origin of the bi-exponential behavior in the case of organic-inorganic perovskites PL is still debated but the fact that the present samples exhibit a similar kinetic behavior hints to a direct connection between the X-ray transients and the PL.

**TABLE I.** Parameters of the biexponential fit of the kinetic traces of Figure 2.

Sample	τ_1_(A_1_)	τ_2_(A_2_)
CsPb(ClBr)_3_	195 ± 25 ps (72%)	132 ± 30 ns (28%)
CsPbBr_3_	542 ± 44 ps (64%)	104 ± 14 ns (36%)

The Pb L_3_-edge spectrum of most compounds is generally rather featureless, e.g., lead oxides with different oxidation states,^66,67,71^ due to the very short core-hole lifetimes, which broaden all transitions, making their assignment difficult. Using high energy resolution fluorescence detection (HERFD) X-ray absorption near-edge spectroscopy (XANES), which exploits the apparent reduction in the core-hole lifetime broadening, Glatzel and co-workers^67^ could unravel more details of the Pb XANES in various Pb^2+^-containing compounds, in particular, PbO, whose Pb L_3_-edge XANES and HERFD spectra are reproduced in Figure S10 (supplementary material) and compared with the XANES spectrum of our sample. The HERFD spectrum of PbO reveals three features at 13.032 keV, 13.042 keV, and 13.054 keV, which are also visible in the TFY XANES spectrum. However, our spectrum does not show such clear-cut features, which are anyway expected to lie at different energies since it is a different compound. The analysis of the spectra in Ref. 71 was carried out by simulating the density of states in order to reproduce the spectra. It was concluded that the pre-edge feature at 13.032 keV is dominated by the Pb p-orbitals, but it arises in the L_3_-edge spectrum due to their strong mixing with Pb d-orbitals. At higher energies, the spectrum is dominated by the Pb d-orbitals, with a weak contribution of Pb p-orbitals. Interestingly, O-ligand orbitals also contribute to the 13.032 keV and 13.054 keV features in the PbO spectrum.

The L_3_-edge transients in Figure 3 cannot be interpreted in terms of a chemical edge shift, which would be caused by a change of oxidation state of the Pb^2+^ ions, turning them to Pb^+^ centres, and leading to an edge shift to lower energies. This can be ruled out based on Figure S12 (supplementary material) where we show calculated difference spectra as a function of the edge shift from −6 eV to +6 eV. Rather, the decreased intensity right at the edge (i.e., above the Fermi level) is due to the fact that upon laser excitation, electrons are transferred to the CB (i.e., above the Fermi level), filling the Pb 6p and 6d orbitals, where they remain delocalized at ≥80 ps time delays, thus reducing the transition probability as a result of the lower density of unoccupied states. Fully delocalized charges in the CB imply that the oxidation shift of the edge is negligible. Concerning the positive features around 13.065 keV, structure changes may well cause it, which is expected if polaron formation occurs around the Br^0^ atoms. On the other hand, the appearance of the positive feature at 13.040 keV in the chlorinated sample points to an electronic effect. Since the density of unoccupied states of Pb has decreased after excitation, a positive feature in this region most likely results from hybridization with Cl or Br-ligand orbitals,^36,43^ which have been depopulated by photoexcitation.

Finally, the transient signal at the Cs L_2_-edge shows no signature of an electronic (at the edge) or structural (above the edge) change. The lack of an electronic signature may be rationalized by the fact that in both organic and inorganic perovskites, the cation orbitals are far above the bottom of the CB,^8,36,41–48^ and therefore, they would not contribute to the charge carrier dynamics at time delays ≥80 ps. However, the exact energy of these orbitals is not known precisely, and given that our pump energy is ∼1.2 eV above the CB minimum in the case of the purely brominated sample, one may not exclude that a transient signal shows up at earlier times. As far as the lack of signature in the above edge region is concerned, we note that already the steady-state ground state spectrum is featureless in this region and is rather insensitive to structure. We conclude that the cation is not a direct player in the fate of the charge carriers, at least for time scales >80 ps.

There is an on-going debate about electron and hole mobilities in organic-inorganic perovskites,^15,16,18,19^ while only one theoretical study exists for inorganic ones.^79^ As far as the organic-inorganic ones are concerned, Zhu and Podzorov^18^ have suggested that charge carriers may be protected by the formation of a large (heavy) polaron, which could also explain the modest charge carrier mobilities. Brenner *et al*.^19^ have suggested that the relatively (compared to more conventional semi-conductors) low mobility of charge carriers is due to a strong electron-phonon coupling. They argued that since the mobility is proportional to the carrier scattering time and inversely proportional to the effective mass, and since effective mass values of organic-inorganic perovskites are similar to those of common inorganic semiconductors, then the mobility must be limited by scattering, which can either be due to electron-phonon coupling or impurity scattering. They suggest that the electron-phonon coupling can also explain the long lifetimes of the charge carriers. The small hole polaron formation inferred from our results is compatible with a strong electron-phonon scattering. These considerations concern the organic-inorganic perovskites but carrier mobilities appear to be significantly higher in inorganic ones, which hints to different charge transport properties.^7,80,81^ Using the hybrid density functional theory, Neukirch *et al.*^79^ found that in a CsPbI_3_ cluster electron and hole polarons are formed, with a larger BE for the electrons. This seems to fit with the conclusion of a weakly localized exciton in organic-inorganic perovskites proposed by He *et al*.,^30^ which they attributed to band tail states, mainly due to electrons at the bottom of the CB. Our results suggest that on the contrary for the present inorganic perovskites, it is the hole that is localized rather than the electron. This debate about the charge carrier mobilities of perovskites calls for more detailed investigations.

## V. CONCLUSIONS

We have investigated the fate of the charge carriers in photoexcited inorganic pure (Br) and mixed halide (Br-Cl) perovskites nanoparticles in solution using picosecond X-ray absorption spectroscopy. The main conclusions are that at times ≥80 ps, holes are fully localized at Br atoms, forming small polarons, while electrons remain delocalized in the CB. The Cs cation does not show any effect upon photoexcitation, leading to the conclusion that it does not play a role in the charge transport and localization, at least at times >80 ps. The decay of the Br K-edge transient signal is bi-exponential, showing the same trends as the optical PL of organic-inorganic compounds, with the long component being of the same order of magnitude. The results are identical for purely brominated systems, and for chlorinated ones, however, some subtle differences appear in the decay times of the Br K-edge transient and at the Pb L_3_-edge transients, which call for further studies.

On a broader perspective, the present work highlights the benefits of a time-resolved element-selective tool for the study of charge carrier dynamics in multi-element materials. While time-resolved XAS was previously used to study photoexcited solids such as TiO_2_,^2,61^ it mainly detected trapping of charge carriers at defects. In the present work, we probed with atomic-selectivity for the first time, the time evolution of the atomic actors involved in the fate of the charge carriers in a multi-element semi-conducting solar material where the role of defects is minimized. Extensions of such experiments into the femtosecond time domain are being planned and will deliver insight about the charge transport and the time scale of localization, which are crucial for improving the performances of such materials.

## SUPPLEMENTARY MATERIAL

See supplementary material for the description of the chemical synthesis (with TEM and powder diffraction figures) and of the experimental procedures for time-resolved X-ray absorption spectroscopy. X-ray and optical absorption spectra before and after the measurements to check for the sample photostability. Pump fluence dependence of the Br K-edge transient signal. Simulated transient difference spectra at the Br K-edge and the Pb L_3_-edge.
